# Progress towards Millennium Development Goals 4 & 5: strengthening human resources for maternal, newborn and child health

**DOI:** 10.1186/1472-6963-15-S1-S1

**Published:** 2015-06-08

**Authors:** Jean Christophe Fotso, Linda Fogarty

**Affiliations:** 1 Concern Worldwide US, New York, NY, USA; 2 Jhpiego, Baltimore, MD, USA

## 

Since the 1990s, developing countries as a whole have made remarkable efforts to reduce under-five mortality rate from 90 to 48 deaths per thousand live births in 2012. As a result, the number of annual under-five deaths dropped by nearly 6 million during that period [1,2]. Unfortunately, the pace of reduction was slowest in sub-Saharan Africa, making it impossible that the region will reach the target for the Millennium Development Goals (MDGs) 4 [1], but a few countries, including some of the poorest such as Ethiopia, Liberia, Malawi and Niger, have already met the MDG 4 target, or are making progress that are consistent with the target [3]. At the same time, global maternal deaths have been nearly halved since the 1990s, from 380 deaths per 100,000 live births to 210 in 2013. In sub-Saharan Africa and South Asia, the two regions where more than 85% of all maternal deaths occur, the maternal mortality ratio (MMR) dropped from 990 to 510, and from 530 to 190, respectively [2]. Skilled birth attendance (SBA), one of the proximate determinants of maternal mortality, increased only modestly during the period 1990-2012, from 40% to 53% in sub-Saharan Africa, and from 33% to 51% in South Asia [2,4]. As with most health indicators, there are wide variations in the coverage of SBA from 20% or less in Ethiopia and Niger, to more than 80% in countries including Benin, Namibia and Botswana [3].

Most maternal, newborn and child deaths result from causes that can be adequately treated by a package of well-known interventions [5-7]. One of the main challenges in delivering essential and life-saving interventions to avert these deaths is the shortage of a well-trained, supervised, and motivated health workforce [8,9]. With the vast amount of evidence showing that increased availability of skilled health workers is directly linked to improved maternal, newborn and child health (MNCH) outcomes, health workforce development is increasingly seen as a critical component of MNCH care delivery [10].

## Human resources for MNCH and progress towards MDGs 4 & 5: what have we learned?

In the decade since the World Health Report’s call to action on human resources for health (HRH) [11], tremendous global attention has been placed on strengthening human resources for MNCH, notably by increasing the sheer number of health providers and improving the quality of MNCH care they provide. Though the density of skilled providers falls below the World Health Organization (WHO) threshold for obtaining adequate MNCH care of 23 per 10,000 people in 53 of the 68 priority countries [9] the recent attention on HRH has shed light on ways to ensure that the right health workers are in the right place providing the right care for women and children.

### The right health workers

Though it is well-recognized that having a skilled birth attendant (SBA) is one of the most reliable predictors of positive maternal and neonatal health outcomes, policies supporting SBAs in the workforce are often lacking. For example, in Africa, where the global health crisis is most severe, very few national HRH policies exist to guide the training and deployment of the health workforce for maternal, newborn and child health [12]. Where policies are in place, the divide between policy and practice, including imbalances in the workforce structure and distribution, stands in the way of equitable service access, suggesting the need for a more strategic approach to health workforce management [13]. One promising tool to understand and begin to address this divide is a task analysis of the current national MNCH workforce, a method in use in Mozambique to streamline the workforce [14]. Such approaches are critical first steps towards developing the proper policies and plans required to improve the effectiveness of the public health workforce in a sustainable way [15,16].

Once the right type of worker is determined for the country’s needs, recruiting the right candidates and educating them in the right manner--providing up-to-date competency-based education with adequate mentored practice--is critical. With significant increases in donor funding for pre-service education (PSE), evidence in this area has grown in recent years. Johnson and colleagues developed a conceptual model of the health impacts of PSE and collected evidence of the effectiveness of common PSE input. They also highlighted the essential role of clinical preceptors, the importance of professional regulation and the effectiveness of targeted recruitment of students from rural and low-resource settings to improve health worker retention [17].

### …In the right place

Even in countries where sufficient numbers of providers are available in urban centers, severe shortages exist in rural areas. In Guinea, for example, where fewer than half of nurses and a fifth of midwives needed for MNCH care are currently available, supply is lowest in rural areas, and demand is expected to grow by 22% in the next decade [18]. In countries where home births are still the norm, one promising method for providing women with the care they need is through community health providers. One recent analysis summarizing community-centric methods to reduce postpartum hemorrhage (PPH) reported that across 18 programs, a total of 6,732 women took misoprostol—a uterotonic that does not require refrigeration—and that distribution by community health providers more than doubled coverage rates compared with health workers and ANC providers alone [19].

Another way to ensure that critical services are available where women need them is by retaining the skilled maternal health providers currently in place, by improving job satisfaction and formal supervision systems, for example [20].

### …Providing the right care

Making the most of the current workforce to meet demands is essential in addressing the HRH crisis for MNCH care. Task-shifting has improved access and quality of care in a variety of settings [21-23]. For example, in Mali, trained matrones—or auxiliary midwives--were as skilled at performing active management of the third stage of labor (AMTSL) as their SBA colleagues, which led to a shift in national policy [24]. Though task-shifting may not be the entire answer to the health workforce crisis, it certainly is part of the solution [25].

Quality and coverage of interventions known to improve maternal and newborn health vary widely. For example 90% of women have at least one ANC visit, but only 30% of babies receive a postnatal (PNC) visit [26]. A systematic review found that staff shortages were the most consistent barrier to quality emergency obstetric care [27]. Though increasing health worker numbers and quality--“scaling up” and “skilling up”--are both needed, Fauveau, Sherratt and de Bernis encouraged prioritizing quality over numbers, and shared a framework for addressing scaling up quality midwifery at the community level [28].

The most common “go-to” intervention to improve provider performance is in-service training. Though training is essential to develop the health workforce, Gaye and Nelson argue that training alone cannot succeed without addressing common pitfalls, including uncoordinated training, training the wrong people, taking workers from their jobs to be trained, and inattention to other factors needed to support workers’ new skills, such as organizational support and supportive supervision [29]. Another recent review of in-service training approaches reports that multiple learning modalities, clinical simulations, repetition, and practice with feedback are effective techniques, while passive instruction, such as reading and lecture, have limited effect [30].

Fritzen (2007) proposed using a lens to look at what the workforce ‘can do’ and what it ‘will do’ to help improve service quality, citing clear terms of employment, supervision, employee recognition, incentives to improve motivation, and performance management techniques [12]. Quality improvement approaches have been widely used with some success, such as in Tanzania, where performance in newborn care improved, but newborn resuscitation performance remained unchanged [31].

## Overview of the Supplement

Drawing largely from the evaluation of HRH projects implemented as part of Concern Worldwide US’ *Innovations for MNCH*, this Supplement seeks to expand the evidence base on the critical role of human resources for MNCH in countries’ efforts towards the MDGs 4 & 5 targets. The Supplement includes four core papers. The first paper by Weiss et al. examines if supervision of Care Group (a community-based implementation strategy for the delivery of social and behavior change interventions) activities by the Burundi Ministry of Health (MOH) personnel could achieve the same child health outcomes as supervision provided by specialized non-governmental organization (NGO) staff. Drawing on quantitative data from the evaluation which had a quasi-experimental design, the authors show that the MOH-led Care Group model performed at least as well as the NGO-led model in achieving specific child health and nutrition outcomes. This finding suggests increased potential for sustainability.

The second paper by Vesel at al. is based on the evaluation data of a project named Helping Health Workers Cope (HHWC), which was implemented in a rural district of Sierra Leone. The paper shows that the intervention had a positive effect on coping skills, stress levels and provider-provider and provider-client relationships, and observed associations between changes over time in coping skills and changes in relationships. The authors argue that integrating psychosocial counseling and training interventions into health worker pre-service and in-service curricula would allow the positive effects of this intervention to be expanded across other areas of Sierra Leone.

The third paper uses data from a somewhat similar project, entitled Quality Circles, which focused on interventions for both formal health workers and traditional birth attendants. Authored by Higgins-Steele et al., its results show that the intervention had positive effects on organizational skills and relationships. They also indicate that improvements over time in organizational skills variables – problem-solving, strategizing and negotiation skills – were strongly associated with a change in some of the relationship variables. The authors conclude that this approach of bringing together peers in a structured process of group work and individual skill development is critical in low-resources contexts, where active participation and resourcefulness of health workers can contribute to better health service delivery.

The fourth paper by Fotso et al. shifts the geographic focus from Africa to rural India, specifically in the context of community-based MNCH service delivery by female Community Health Workers (CHWs), known as Accredited Social Health Activists (ASHAs). Examining the influence of a male engagement project on the utilization and community-based delivery of MNCH care in a rural district of India, the authors found gender-based divisions of work and space in core areas of delivery and use of MNCH services. The study ultimately unveils the complementarity of male and female CHWs in the community-based delivery of, and increased demand for, MNCH services.

Finally, the concluding commentary by Lehmann casts the four papers in the broader context of human resources strengthening for health systems resilience. The author suggests that the interventions reviewed by this set of papers be viewed along a continuum of program strengthening and systems resilience: repairing damage caused in the past, strengthening systems going forward, and introducing new actors by extending traditionally female-dominated community-level care to include men. The commentary ends with critical questions related to scale up and sustainability.

## About the Innovations for MNCH initiative

The *Innovations for Maternal*, *Newborn and Child Health* is an Initiative of Concern Worldwide US. It seeks to develop and test innovative interventions and strategies to address common barriers that prevent essential health services from reaching women and children. It is supported by a multi-year grant from the Bill & Melinda Gates Foundation. The initiative started in 2010 with comprehensive research on the most pervasive barriers to the delivery of MNCH services, and then generated intervention ideas to address those barriers. The process engaged communities in low-resource settings, tapped unheard or unconventional voices at both global and country levels, and honed the ideas through cross-disciplinary inputs from experts from many different fields. A first set of projects (referred to as Phase I) were designed, implemented and evaluated between 2009 and 2013 (See Additional file 1). From the initial basket of ideas, the learnings from Phase I, and additional formative research in selected countries, five additional projects were designed and launched in 2013 and 2014 (see Additional file 2).

The *Innovations for MNCH’s* research, monitoring and evaluation (RME) plan aims to determine the effectiveness of the interventions in overcoming the identified barriers to MNCH services, and to explore how and why the interventions achieved their results. Data sources include routine monitoring, baseline and endline assessments and process documentation. Also important to the initiative’s overall strategy is the experimentation and learning related to the application of human-centered design in MNCH in developing country settings [32]. Another novelty associated with *Innovations for MNCH* is the incorporation of human rights principles into project and research designs. Drawing from major UN frameworks and with inputs from UNICEF and external experts, *Innovations* developed its human rights framework around four principles: right to health, non-discrimination, participation, and accountability [33,34]. These research components are being used to inform new approaches to program design, generate evidence about the effectiveness of the interventions, and shed light on the potential of human-centered design and the application of human rights principles in MNCH.

## Competing interests

The authors declare no competing interests.

## Authors contributions

JCF and LF conceptualized the study, wrote the manuscript, and approved the final version.

## Supplementary Material

Additional file 1 Annex 1 Click here for file

Additional file 2 Annex 2 Click here for file

## Declarations

The publication costs for this article were funded by the *Innovations for Maternal*, *Newborn & Child Health*, an initiative funded by Bill & Melinda Gates Foundation.

This article has been published as part of *BMC Health Services Research* Volume 15 Supplement 1, 2015: Improved access to maternal, newborn and child health services: strengthening human resources for health. The full contents of the supplement are available online at http://www.biomedcentral.com/bmchealthservres/supplements/15/S1.
