# Supplementary material for: Progress towards Millennium Development Goals 4 & 5: strengthening human resources for maternal, newborn and child health
Source: BMC Health Serv Res. 2015 Jun 8;15(Suppl 1):S1. doi: 10.1186/1472-6963-15-S1-S1 (PMC4464219; doi:10.1186/1472-6963-15-S1-S1)
Supplement: Additional file 1 — Annex 1 [file 1472-6963-15-S1-S1-S1.docx]

**Annex 1**. List of Innovations for MNCH’s Phase I (completed) projects

| **Project and country of implementation** | **Overview** |
| --- | --- |
| ICT for MNCH, Malawi | Using information and communications technologies to connect pregnant women, caretakers and children to health workers, thereby giving them immediate access to personalized health advice, tips and reminders via individual and community phones. |
| Male Health Activists, State of Odisha, India | Building on existing networks of female community health workers by adding a cadre of Male Health Activists who promote the importance and benefit of MNCH services among male household decision-makers. |
| Helping Health Workers Cope, Sierra Leone | Providing access to group and individual counseling services for health workers to cope with the stress and pressures of their work as well as providing training sessions for these health workers on stress management, self-care and client care. |
| Quality Circles, Sierra Leone | Supporting health workers and traditional birth attendants to develop peer learning, peer support and joint problem solving skills to empower them to be more effective in their jobs and resolve gaps and failures in the health system. |
