# Supplementary material for: Progress towards Millennium Development Goals 4 & 5: strengthening human resources for maternal, newborn and child health
Source: BMC Health Serv Res. 2015 Jun 8;15(Suppl 1):S1. doi: 10.1186/1472-6963-15-S1-S1 (PMC4464219; doi:10.1186/1472-6963-15-S1-S1)
Supplement: Additional file 2 — Annex 2 [file 1472-6963-15-S1-S1-S2.docx]

**Annex 2**. List of Innovations for MNCH’s Phase II (ongoing) projects

| **Project and country of implementation** | **Overview** |
| --- | --- |
| Essential Newborn Care Corps (ENCC), Sierra Leone | Rebranding traditional birth attendants as Maternal Newborn Health Promoters who provide health advice and referrals to the health facility for pregnant women and newborns during home visits while selling health-related products. |
| Care Community Hub (CCH), Ghana | Improving motivation and job satisfaction among frontline health workers through a mobile “app” which offers opportunities for professional development and reduces the sense of social isolation among health workers posted in remote areas. |
| Community Benefits Health (CBH), Ghana | Creatively using non-monetary incentives to cultivate communities’ investment and commitment to supporting and improving maternal and child health. |
| Maker Movement for MNCH (Maker), Kenya | Creating a hub that links local makers, biomedical engineers and MNCH practitioners to design, prototype and test low-cost, high- quality, open-source and locally produced essential medical equipment, devices and spare parts. |
| Mobile Urgent Maternity Service (MUM), Kenya | Using computer modeling to determine ideal locations of ambulances and static emergency obstetric and neonatal care clinics, and to create optimal routes for mobile clinics. |
